# Investigation of the microbial communities colonizing prepainted steel used for roofing and walling

**DOI:** 10.1002/mbo3.425

**Published:** 2016-12-20

**Authors:** Tran T. Huynh, Ili Jamil, Nicole A. Pianegonda, Stephen J. Blanksby, Philip J. Barker, Mike Manefield, Scott A. Rice

**Affiliations:** ^1^ The Centre for Marine Bio‐InnovationThe University of New South WalesSydneyNSWAustralia; ^2^ The School of Biotechnology and Biomolecular SciencesThe University of New South WalesSydneyNSWAustralia; ^3^ ARC Centre of Excellence for Free Radical Chemistry and BiotechnologySchool of ChemistryUniversity of WollongongWollongongNSWAustralia; ^4^ BlueScope InnovationPort KemblaNSWAustralia; ^5^ Central Analytical Research FacilityQueensland University of TechnologyBrisbaneQLDAustralia; ^6^ The School of Biological, Earth and Environmental SciencesThe University of New South WalesSydneyNSWAustralia; ^7^ The Singapore Centre for Environmental Life Sciences Engineering and the School of Biological SciencesNanyang Technological UniversitySingapore

**Keywords:** biofilms, biofouling, building materials, environmental exposure, microbial communities

## Abstract

Microbial colonization of prepainted steel, commonly used in roofing applications, impacts their aesthetics, durability, and functionality. Understanding the relevant organisms and the mechanisms by which colonization occurs would provide valuable information that can be subsequently used to design fouling prevention strategies. Here, next‐generation sequencing and microbial community finger printing (T‐RFLP) were used to study the community composition of microbes colonizing prepainted steel roofing materials at Burrawang, Australia and Kapar, Malaysia over a 52‐week period. Community diversity was low and was dominated by *Bacillus* spp., cyanobacteria, actinobacteria, *Cladosporium* sp., *Epicoccum nigrum*, and Teratosphaeriaceae sp. Cultivation‐based methods isolated approximately 20 different fungi and bacteria, some of which, such as *E. nigrum* and *Cladosporium* sp., were represented in the community sequence data. Fluorescence in situ hybridization imaging showed that fungi were the most dominant organisms present. Analysis of the sequence and T‐RFLP data indicated that the microbial communities differed significantly between locations and changed significantly over time. The study demonstrates the utility of molecular ecology tools to identify and characterize microbial communities associated with the fouling of painted steel surfaces and ultimately can enable the targeted development of control strategies based on the dominant species responsible for fouling.

## 1 Introduction

Microorganisms in the environment have the ability to colonize almost all surfaces, including manmade surfaces (Rosado, Mirao, Candeias, & Caldeira, 2014). Colonization by these microbial communities can cause degradation of various materials leading to aesthetic and structural damage. Such effects range from pigment‐based discoloration to staining and even deterioration of structural integrity through microbially induced corrosion of metal‐ and concrete‐based materials (Ciferri, 1999). Microorganisms are capable of colonizing a wide variety of natural surfaces, and are therefore found on a broad spectrum of manmade products, including paintings, wood, paper, metals, and concrete structures (Blanchette et al., 2004; Michaelsen, Pinzari, Ripka, Lubitz, & Pinar, 2006; Stephen & MacNaughton, 1999). The colonization of the walls and air‐conditioning ducts of homes and offices is associated with ‘sick building syndrome’ where the colonizing organisms contribute to the ill health of humans (Burge, 2004; Skov & Valbjorn, 1987; Wargocki, Wyon, Sundell, Clausen, & Fanger, 2000). The colonization of interior and exterior surfaces is unsurprisingly associated with a wide range of microbes and in most cases can be habitat specific (Dubosc, Escadeillas, & Blanc, 2001; Gaylarde, Ortega‐Morales, & Bartolo‐Perez, 2007; Ortega‐Calvo, Hernandez‐Marine, & Saiz‐Jimenez, 1991).

The microbial community composition depends on several environmental factors, including temperature and humidity (Gaylarde & Gaylarde, 2005), and the physical properties of the surface. For example, fungi were found more than bacteria on painted surfaces (Gaylarde & Gaylarde, 2005) although cyanobacteria, heterotrophic bacteria, and algae were also found on painted surfaces. Algae were more prevalent than cyanobacteria on roofing materials in southeastern and northwestern areas of the United States where it is humid (Gaylarde & Gaylarde, 1999). Fungi and cyanobacteria are able to survive under conditions of high UV exposure and repetitive cycles of desiccation and rehydration (Potts, 1994; Yancey, Clark, Hand, Bowlus, & Somero, 1982) and their survival under adverse environmental conditions is linked to their ability to form biofilms (Gorbushina et al., 2004). Biofilm formation has been shown to increase the tolerance of communities to stress (Burmolle et al., 2006; Lee et al., 2014) and this includes protection from desiccation (Flemming & Wingender, 2010) where the hydrated biofilm matrix retains water necessary for the viability of the community.

Once microorganisms have colonized a surface, they can alter the properties of the materials through a number of different processes. The substratum may, for example, be oxidized by the production of organic acids (Gu, Ford, Berke, & Mitchell, 1998) as well as nitric and sulfuric acids (Sand, 1997; Sand & Bock, 1991) and the production of such acids along with other metabolic by products may also cause corrosion (Boopathy & Daniels, 1991; Hamilton, 1985). The rate of corrosion has been shown to be considerably higher with mixed microbial cultures than for pure cultures and most fouling in environmental settings involves microbial communities rather than isolated populations (Beech & Sunner, 2004). In addition to surface deterioration, the accumulation of biomass can result in the appearance of dark staining (Berdahl, Akbari, Levinson, & Miller, 2008). This has significant impacts on cultural and historical items such as paintings, sculptures and monuments. Damage or discoloration caused by colonization on materials may result in failure of the materials, deterioration of function, and customer dissatisfaction, all of which have significant economic impacts as well as social impacts, in the case of culturally important items. While largely aesthetic, discoloration of building materials can also alter the engineered properties of such materials. For example, some roofing materials are designed to reflect solar infrared radiation to reduce heat transfer to the interior of the building (Berdahl et al., 2008). Colonization and discoloration inhibit solar reflection and thus retard the beneficial properties of the materials.

Colonization of roofing materials represents a significant challenge for the invading community. Microbial colonization occurs under all weather conditions, including extremes of temperature, desiccation, intermittent nutrient loading, and periods of intense UV exposure (Berdahl et al., 2008). Such habitats may also be relatively limited in nutrients as they are designed for water runoff and do not accumulate environmental materials on their surfaces. Despite representing a relatively extreme habitat, bacteria and fungi can colonize the roofing materials, resulting in the consequences described above. Some microorganisms adapt to such adverse habitats by forming resistant structures, such as spores, which can rapidly resume vegetative growth when conditions become favorable again (Nicholson, Munakata, Horneck, Melosh, & Setlow, 2000; Willetts, 1971). Additionally, some organisms produce pigments that are protective from the UV and such pigment production also contributes to the aesthetic issues associated with fouling (Quesada & Vincent, 1997; Rossi et al., 2012). While the problem of microbial infestation of building roofs is well known, there is little information on the communities involved as well as their impacts on such materials.

To characterize the microbial communities on roofing materials and how such communities change over time, molecular techniques including community sequencing, T‐RFLP, and fluorescence in situ hybridization (FISH) techniques were used. Panels of prepainted steel sheeting were deployed at two locations, Burrawang (Australia) and Kapar (Malaysia), on exposure racks and were analyzed at monthly intervals over a period of 12 months. This was performed as two separate time series at the Burrawang site, the first for 12 months and a second for 6 months, deployed 1 year after the first series was exposed. The correlation between environmental factors and the microbial communities that formed over time was also investigated. The results indicated that these materials were colonized by communities characterized by 216 fungal and 562 bacterial species, but that were primarily dominated by less than 10 species of each domain. When the two sites were compared, the composition of fungi and bacteria were significantly different and they also change over time. While there were clear patterns in community change over time, environmental factors, such as rainfall and temperature, seemed have no clear effect on the community structure.

## 2 Experimental Procedures

### 2.1 Study site and sampling

The samples were collected monthly over 52 weeks in triplicate at Burrawang, NSW, Australia (34.6000°S, 150.5167°E) and Kapar, Malaysia (3.1397°N, 101.3678°E). The Burrawang panels were 9.5 cm × 23.5 cm and the Kapar panels were 11 cm × 13 cm (Fig. S2). Both types of panels were mounted on exposure racks consisting of a series of horizontal wooden slats to which panels are attached by placing them between two rubber washers, tightened with a screw. Coupons with diameter 2.8 cm were punched out from the panels for microscopic observation. The exposure study was repeated in the first half of the subsequent year at Burrawang, NSW.

### 2.2 Temperature and rainfall data collection

To determine the impact of rainfall and temperature, the daily maximum temperature and rainfall data were collected and averaged for the 28 day before the panels were collected. Moss Vale Australian Weather Station (AWS) was used for temperature data, as it is closest to Burrawang. Rainfall data was collected from Range Street, Burrawang. All data were extracted from the Bureau of Meteorology website (http://www.bom.gov.au/climate/data/). Temperature and rainfall in Kapar, Malaysia were collected from the Selangor State from Jabatan Meteorologi Malaysia (www.met.gov.my).

### 2.3 Cultivation and isolation

Fungi and bacteria were isolated from samples by swabbing the sample surfaces and incubating on malt extract agar (fungi) and R2A agar (bacteria) as general cultivation media. The agar plates were incubated at room temperature for 3–5 day until growth was observed. Single colonies were then collected on a fresh agar plate for isolation.

### 2.4 Fluorescence in situ hybridization

After the samples were transferred to the laboratory, the coupons were fixed overnight with 4% paraformaldehyde, and kept at 4°C until use. Fixed coupons were then dehydrated in a series of ethanol at room temperature: 50%, 80%, and 96% for 3 min each and air‐dried. Hybridization was performed in a hybridization chamber at 46°C in hybridization buffer [0.9 M NaCl, 20 mM Tris‐HCl (pH 7.4), 0.01% sodium dodecyl sulfate (SDS), and 30% formamide] for 3 hr. Fluorescent probes were used at a concentration of 5 ng μl^−1^ labeled probe hybridization buffer [0.9 mol/L NaCl, 20 mmol/L Tris‐HCl (pH 7.4), 0.01% SDS, and 30% formamide]. The oligonucleotide probes (IDT DNA, Iowa) used were EUB338 (GCTGCCTCCCGTAGGAGT) which binds to bacterial ribosomal RNA (Amann, Ludwig, & Schleifer, 1995; Stahl et al., 1989) and EUK516 (ACCAGACTTGCCCTCC) which binds to the fungal ribosomal RNA (Amann et al., 1995). Probes EUB338 and EUK516 were labeled with the fluorochromes, indocarbocyanine (Cy3), or indodicarbocyanine (Cy5), respectively. The coupons were incubated in 100 μl of hybridization mixture of oligonucleotides, then carefully rinsed and further incubated at 48°C in wash buffer [2 mmol/LTris‐HCl, 5 mmol/L EDTA, 0.01% SDS and a variable amount NaCl to optimize probe specificity] for 20 min. The wash buffer was then carefully rinsed off with Milli‐Q^®^water, the slides were air‐dried in the dark and stained with 1 μg ml^−1^ of 4′6‐diamidino‐2‐phenylindole (DAPI) solution for microscopic observation. Microscopy analysis was performed using an inverted confocal laser scanning microscope (Nikon, C1si) with excitation wavelengths of 408 nm, 561 nm, and 637 nm for DAPI, Cy3, and Cy5 labeled probes, respectively. ImageJ software version 1.36b (http://rsb.info.nih.gov/ij/download.html) was used to estimate the proportion of bacteria (stained with Cy3) and fungi (stained with Cy5) against total microorganisms (stained with DAPI). The analysis was performed on fives images from different locations of the coupons in triplicate.

### 2.5 DNA extraction from the panels and microbial cultures

Total genomic DNA was extracted using the modified CTAB/phenol method (Cubero, Crespo, Fatehi, & Bridge, 1999; Rogers & Bendich, 1994). An area of 100 cm^2^ of the exposed panels was swabbed using a sterile cotton bud. For bacterial and fungal cultures, overnight cultures grown in LB (bacteria, 37°C) or peptone yeast extract glucose (fungi, room temperature) were pelleted by centrifugation. The incubated cells or swabbed materials were incubated with 20 μl of 200 U ml^−1^ lyticase for 24 hr at 37°C. The samples were incubated with 0.4 ml of 5% CTAB and 0.4 ml of phenol:chloroform:isoamyl alcohol (25:24:1) mixture and placed in the FastPrep bead beater (MP Bio) for 2 min (speed setting 5.5) to lyse cells. Samples were centrifuged at full speed (18,620*g*) for 5 min. The top aqueous phase was transferred to a new tube and mixed with an equal volume of chloroform:isoamyl alcohol, followed by centrifugation at (18,620*g*) for 5 min at 4°C. The top aqueous layer was collected and mixed with two volumes of 30% (w/v) PEG (polyethylene glycol average molecular weight 1,450 g mole^−1^) to precipitate the DNA. This mixture was incubated at 4°C overnight and centrifuged (18,620*g*) for 10 min at 4°C. The pellet was washed with 200 μl of 70% ice cold ethanol, air‐dried for 1 hr, and resuspended in 50 μl of sterile Milli‐Q^®^ water. Extracted DNA was quantified using a Nanodrop^™^ND‐1000 spectrophotometer (Thermo Fisher) and visualized on 1% agarose gel.

### 2.6 PCR amplification

For T‐RFLP and colony sequencing, PCR amplification of the V3 region of the 16S rDNA gene (for bacteria—forward primer V3F 5′AC GTCCAGACTCCTACGGG 3′, reverse primer V3R 5′TTACCGCGGCTGCTGGCAC 3′) or the internal transcribed spacer region (ITS1) of the 18S rDNA gene (for fungi—forward primer ITS1F 5′CTTGGTCATTTAGAGGAAGTAA 3′, reverse primer ITS4 5′TCCTCCGCTTATTGATATGC 3′) was performed. PCR reactions contained 12.5 μl molecular grade water, 12.5 μl of EconoTaq^™^ Plus Green 2× Master Mix (Lucigen), 0.75 μl of each forward and reverse primer (0.3 μmol/L concentration), and 1.0 μl DNA template in 25 μl total volume. Reaction conditions were initial denaturation at 95°C for 4 min, followed by 30 cycles of 30 s at 95°C, 30 s at 55°C, 60 s at 72°C, and a final extension for 10 min at 72°C. PCR products were visualized on a 1% agarose gel. For T‐RFLP, the PCR reaction was performed in triplicate and pooled prior to restriction digestion and subsequent analysis (described below).

### 2.7 Illumina sequencing and data analysis

Illumina sequencing was performed by the Research and Testing Laboratory (Lubbock, TX) using an Illumina MiSeq instrument. PCR amplification and sequencing of the 16S and 18S rRNA genes used the primers 28F (5′ GAGTTTGATCNTGGCTCAG 3′)–519R (5′ GTNTTACNGCGGCKGCTG 3′) that target conserved regions flanking the V1–V3 region of the 16S rRNA gene in bacteria and primers (5′ TGGAGGGCAAGTCTGGTG 3′, 5′ TCGGCATAGTTTATGGTTAAG 3′) for the 18S rRNA gene of fungi (Hume et al., 2012). Data were quality filtered, and processed using the Quantitative Insights Into Microbial Ecology QIIME v 1.5.0‐dev pipeline(Caporaso et al., 2010). Bacterial and fungal sequences were assigned into groups based on their barcodes. The sequences were clustered into operational taxonomic units (OUTs) by de novo clustering using UCLUST reference‐based algorithm (Edgar, 2010). Clustering was conducted at the 97% identity level using the Silva database version 104 (Pruesse et al., 2007). The most abundant sequence from each OTU was selected as a representative sequence for that OTU and taxonomy of that OTU was assigned via the Silva database version 104 and GenBank (http://www.ncbi.nlm.nih.gov/genbank/).

### 2.8 Microbial community fingerprinting analysis by T‐RFLP

For genomic DNA extracted from the panel surfaces as above, the V3 region of the bacterial 16S ribosomal subunit and the ITS region of the fungal 18S ribosomal subunits were amplified. PCR amplification was performed in triplicate with the primers ITS1F and ITS4 (for fungi) (White, Bruns, Lee, & Taylor, 1990), and V3R and V3F (for bacteria); PCR was performed as described above. The forward primers of V3 and ITS1‐4 region were fluorescently labeled with 6‐FAM. PCR products were purified using a DNA clean and concentrator kit (Zymo Research Corporation) and 200 ng of purified PCR products were digested using 5 U of the *Hae*III restriction endonuclease (NEB Biolab) for 4 hr at 37°C, followed by denaturation of the restriction enzyme at 65°C for 20 min. Subsequently, 20 ng of purified digested products were submitted to the Ramaciotti Centre for Genomics, UNSW using an Applied Biosystems 3730 DNA analyzer.

### 2.9 Analysis of T‐RFLP profiles

The T‐RFLP profiles of the FAM‐labeled terminal fragments were analyzed using Peak Scanner V1.0 software (Applied Biosystems, Forster City, CA, USA). A GSLIZ 500 fragment ladder was used for comparison of the T‐RFLPs. The standard range was set from 30 to 550 bp, and fragments less than 30 bp or bigger than 550 bp were eliminated from the analysis. All of the fragments that had a fluorescence unit of 1 or higher were extracted from Peak Scanner and analyzed with the online T‐REX software (available at trex.biohpc.org) (Culman, Bukowski, Gauch, Cadillo‐Quiroz, & Buckley, 2009) to distinguish the true peaks from background fluorescence. The data was subjected to noise filtering and T‐RFLP alignment. Bray–Curtis similarities were calculated, centroid distances were used to average the triplicate samples, and multidimensional scaling (MDS) techniques were used to examine community structure among the panels using PRIMER 6 software.

### 2.10 Statistical analyses

PERMANOVA was used to test the significant differences among the microbial structures among the panels from different incubation sites and time of exposure.

## 3 Results

### 3.1 Microbial biomass on panels fluctuated over time

**Figure 1:**
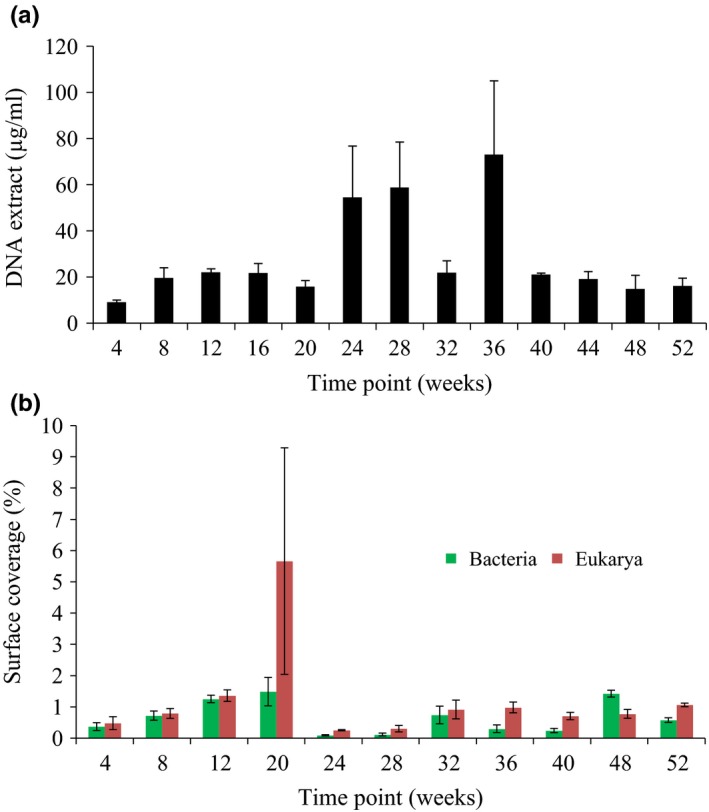
Total DNA extracted from painted steel coupons exposed at Burrawang, NSW Australia, over a 52‐week period (a), error bars represent standard error, *n* = 3. Surface coverage of bacteria and fungi based on FISH images (b), error bars represent standard error, *n* = 15. FISH, fluorescence in situ hybridization

DNA yields from the Burrawang panels over 12 months also suggested an increase in biomass up to 36 weeks followed by a decrease in older panels (Figure 1a). FISH analysis illustrated that there were more fungi than bacteria except at week 48 at Burrawang (Figure 1b and Fig. S1A and S1B). Both bacteria and fungi increased in total surface coverage up to week 20, at which point, there was sudden decrease in total biomass. Subsequently, the biomass increased to approximately 1% surface coverage from week 32 to the end of the experiment. Eight fungi, including *Epicoccum* spp., *Cladosporium* spp., and *Alternaria* spp. and four bacteria, including *Micrococcus* spp. and *Sphingopixis* spp. were isolated from the panels over 12 months (Tables S1 and S2). While the panels were clearly impacted aesthetically at the end of the 12‐month trial, no significant deterioration of the material occurs over this time scale.

### 3.2 The community composition changed over time

To compare the variability in the bacterial and fungal community composition between the Kapar and Burrawang sites, T‐RFLP analysis was performed on biological triplicates of exposure panels from the Burrawang and Kapar sites. The communities changed over time as shown by analysis using the Bray–Curtis similarity algorithm followed by nonmetric multidimensional scaling MDS plots (Clarke, 1993) and PERMANOVA to identify factors responsible for the most diversity between samples. The centroid (the mean lowest dissimilarity among triplicates) distances were used to calculate the average position of the triplicate samples.

**Figure 2:**
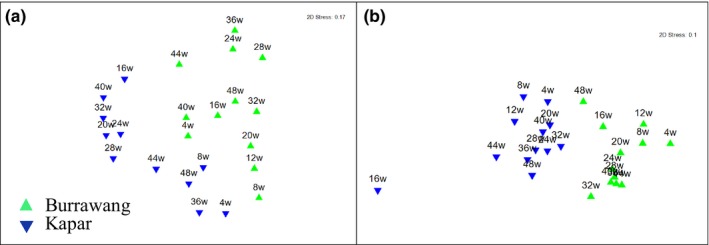
Comparison of fungal diversity on painted steel coupons in two geographically distinct locations, over a 52‐week period. The multidimensional scaling (MDS) plot is based on centroids of triplicate samples from Burrawang (green symbols) and Kapar (blue symbols) based on (a) T‐RFLP profiling or (b) the community sequencing of the ITS region. The week of sample collection is indicated by the number associated with the symbols and comparisons of triplicate coupons were based on the fourth root transformed data using the Bray–Curtis Similarity matrix

**Figure 3:**
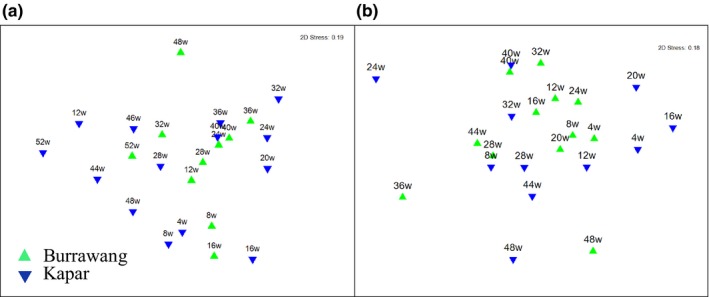
Comparison of bacterial diversity on painted steel coupons in two geographically distinct locations, over a 52‐week period. The multidimensional scaling (MDS) plot is based on centroids of triplicate samples from Burrawang (green symbols) and Kapar (blue symbols) based on (a) T‐RFLP profiling or (b) the community sequencing of the 16S rRNA gene. The week of sample collection is indicated by the number associated with the symbols and comparisons of triplicate coupons were based on the fourth root transformed data using the Bray–Curtis Similarity matrix

The MDS plot showed that fungal communities on the Burrawang and Malaysia panels formed two separate groups during the 52‐week experiment based on both T‐RFLP data (Figure 2a) and sequencing data (Figure 2b). The bacterial compositions at both sites shared more similarity than the fungal communities and this observation was again supported by both the T‐RFLP (Figure 3a) and sequencing data (Figure 3b). At 40 weeks, the bacterial composition was the most similar between the two geographically distinct sites. PERMANOVA analysis of T‐RFLP data showed that time and geographical site accounted for the greatest differences in community composition (*p* = .001) for the fungi (Table 1). The bacterial communities from the Burrawang and Kapar panels were also affected by the exposure time (*p* = .001) but were not affected by location (*p* = .493) (Table 2).

**Table 1:** Comparison of fungal diversity in Burrawang and Kapar samples based on T‐RFLP analysis

Source	*df*	Pseudo‐F	P (perm)
Time	12	2.0841	0.001
Site	1	3.4384	0.008
Time and site	9	2.3523	0.001

**Table 2:** Comparison of bacterial diversity in Burrawang and Malaysian samples based on T‐RFLP data

Source	*df*	Pseudo‐F	P (perm)
Time	12	3.3969	0.001
Site	1	0.93533	0.493
Time and site	9	2.6498	0.001

### 3.3 The fungal communities were dominated by a limited number of operational taxonomic units

**Figure 4:**
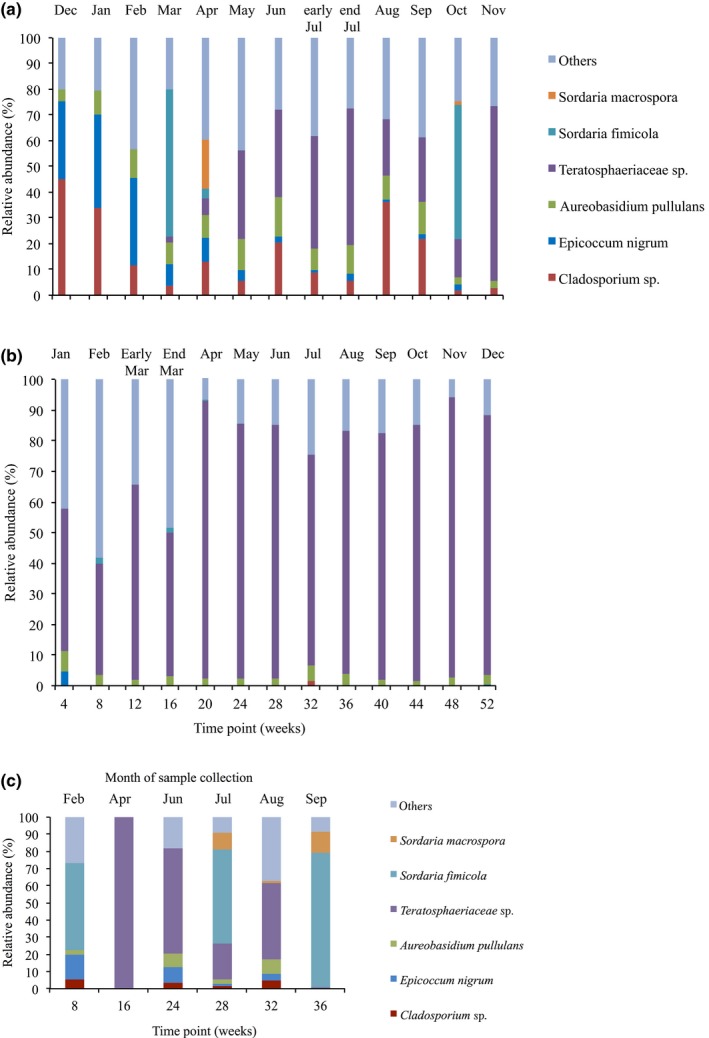
Fungal operational taxonomic units (OTUs) that were present at >1% abundance of the total community for painted steel coupons from (a) Burrawang (time series 1), (b) Kapar and (c) Burrawang (time series 2), as sampled for weeks 4–52. Data represent the average of duplicate samples. The legends for bars shown in (a) are the same for (b) and (c)

There were a total of 287 operational taxonomic units (OTUs) identified for Burrawang time series 1 panels (26 in total) and 216 OTUs identified for the Kapar samples (26 in total). In comparison to the number of OTUs observed for the early (4 weeks) to late (52 weeks) samples, the number of OTUs was constant at 79 for Burrawang time series 1 samples but decreased for Kapar samples from weeks 52 to 55. For the second year of sampling at Burrawang (time series 2), a total of 190 OTUs were identified from seven samples over a period of 36 weeks. The fungal community composition changed over time on all panels, and was dominated by four OTUs that represented up to 90% of the total community at both sites. BLAST analysis using GenBank suggested that these four OTUs corresponded to Teratosphaeriaceae sp. (97% identity)*, Cladosporium* sp. (100% identity), *Epicoccum nigrum* (100% identity), and *Aureobasidium pullulans* (99% identity). For the Burrawang site, *E. nigrum* was present as a relatively high proportion of the community, 30–36%, in the first 3 months (Figure 4a). *Cladsporium* sp. was initially present on the Burrawang panels at a high frequency, but subsequently declined in abundance from months one (44%) to four (3.9%). In contrast, the Teratosphaeriaceae sp. increased from week 20 onward (Figure 4a). This OTU was also found to be highly abundant in the second exposure series, suggesting that this organism is reproducibly an early colonizer of the prepainted metal panels at the Burrawang site (Figure 4c). Approximately 45–80% of the microbial communities from the first 3 months at the Burrawang site were attributed to *Cladosporium* sp. and *E. nigrum*. The Teratosphaeriaceae sp. detected on the Burrawang samples, was also the most abundant species detected in the Kapar samples from the first month, ranging from 40% to 90% of the fungal community (Figure 4b).

**Figure 5:**
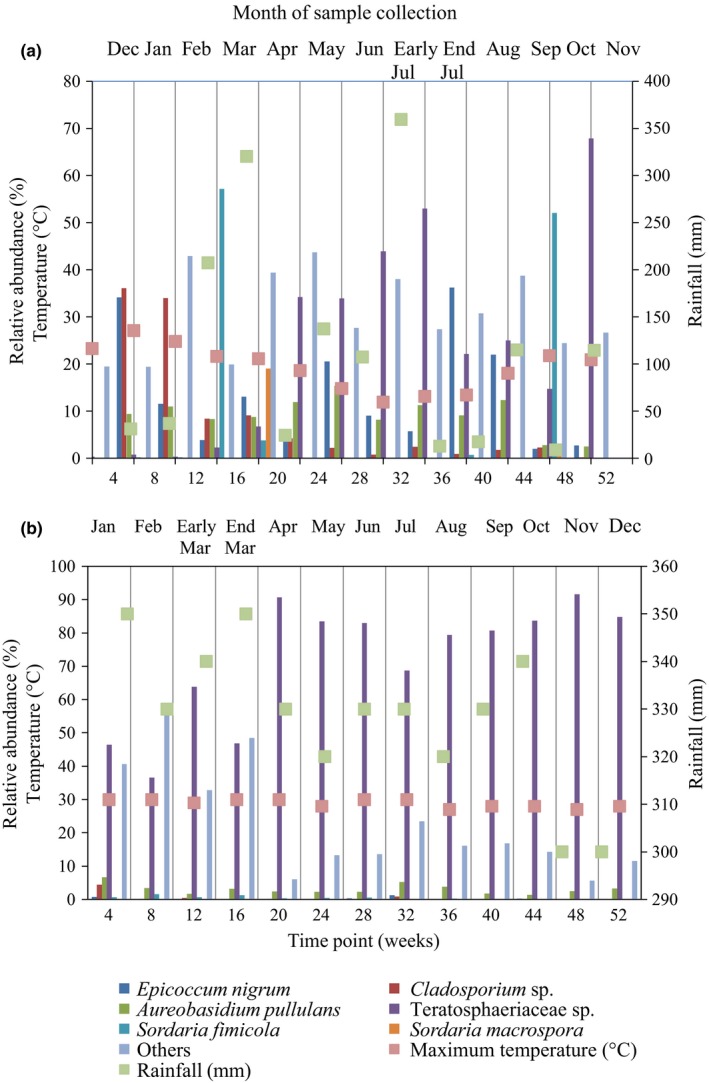
The six most abundant fungi species on (a) Burrawang and (b) Kapar painted steel coupons collected from exposure racks, compared with the maximum temperatures and rainfall. Maximum temperature and rainfall were calculated from the mean of maximum temperature and mean of rainfall of 28 day preceding collection. Operational taxonomic units (OTUs) were determined based on community sequencing of the fungalITSregion

The abundance of *Cladosporium* sp. on the Burrawang panels correlated with temperature (correlation coefficient r = .76) over the 52 weeks incubation period, with a higher abundance in summer compared to winter. There were no other clear relationships between community composition and any of the environmental parameters quantified (Figure 5a and b).

### 3.4 The bacterial community has low diversity

**Figure 6:**
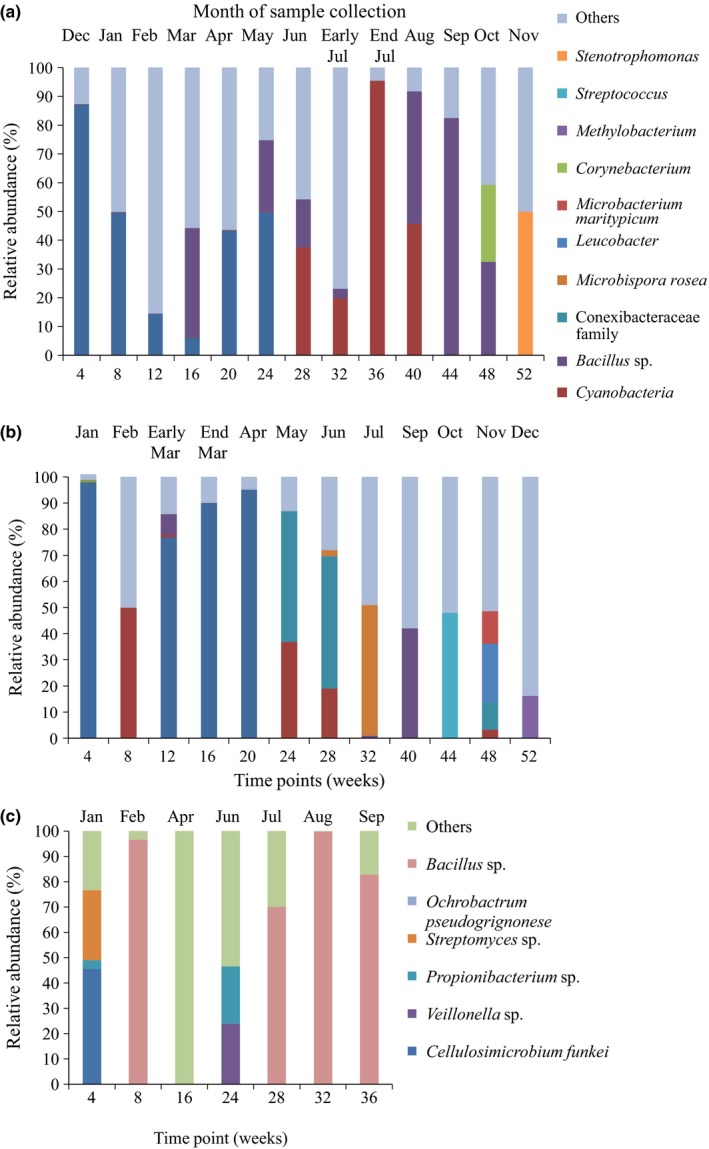
The relative abundance of dominant bacterial species (>1% of the total community abundance) found on exposed, painted steel coupons from (a) Burrawang (time series 1), (b) Kapar and (c) Burrawang (time series 2) based on community 16S rRNA gene sequence data

For the bacterial communities, 562 OTUs were identified for the 52 samples of both sites. For the first set of samples, among these OTUs, 37% were *Bacillus* spp., 15% were cyanobacteria and 43% were actinobacteria, accounting for 97% of the total community. The most abundant OTU observed on the panels collected in the initial stages of the experiment for both Burrawang (time series 1) (Figure 6a) and Kapar (Figure 6b) was assigned to the genus *Cellulosimicrobium*. For the later time points, cyanobacteria were also present in high abundance (Figure 6a). The abundant bacteria found on the second exposure series (time series 2) at Burrawang were *Streptomyces* sp. and Veillonella sp., suggesting that, in contrast to the fungi, the bacterial colonizers were not consistent between years (Figure 6c).

## 4 Discussion

A number of studies have reported that microorganisms contribute to the damage of man‐made structures and materials. However, relatively few of these studies have focused on identifying the species responsible and their patterns of colonization over time. This limitation could be due to the observation that many environmental microorganisms are not readily cultivable in the laboratory. To augment such studies, we have combined culture‐based studies with molecular techniques to investigate the community composition as it forms on painted steel surfaces over time at two geographically separate sites. Based on Illumina MiSeq sequencing of rRNA gene amplicons, the microbial diversity on panels exposed at either Burrawang or Kapar over 1 year were studied and was repeated at Burrawang the following year to determine if the pattern of colonization was reproducible. Over 900 OTUs (>97% identity) were identified based on sequencing, while only approximately different 20 bacteria and fungi species were isolated by cultivation, consistent with the observation that only 0.1%–1% of environmental bacteria are cultivable on standard media under aerobic conditions (Ferguson, Buckley, & Palumbo, 1984). *Cladosporium* sp., *E. nigrum*, and *Aureobasidium pullulans* were the most frequently isolated fungi and this generally agrees with the community sequencing data. Based on the fungal sequencing data, *Cladosporium* sp., *E. nigrum*, and *Aureobasidium* sp. appear to be the initial colonizers of the panels. A comparison of the sequencing data and cultured isolates indicated that these were the only three organisms common to both datasets, and thus, the panels may also be heavily colonized by fungal organisms that were not culturable under the conditions used here. After 30 weeks of exposure, the Burrawang panels were dominated by an OTU that most closely matched Teratosphaeriaceae sp. This group of fungi belong to the class of Dothideomycetes that includes species that inhabit inhospitable niches such as the surfaces of bare rocks (Quaedvlieg et al., 2014). Members of this group have been isolated from eucalypt trees in NSW, Australia (Quaedvlieg et al., 2014) suggesting they are present in the natural environment and this source of the fungus could seed colonization of the panels by transport through the air. Based on the community composition data, future work could seek to recover the some of the more fastidious organisms, such as Teratosphaeriaceae, through the use of more oligotrophic media as opposed to the more general isolation media used here that have typically be used for environmental organisms (Machuca & Ferraz, 2001; Magnusson, Ström, Roos, Sjögren, & Schnürer, 2003; Reasoner & Geldreich, 1985).

Bacteria and fungi commonly coinhabit building materials such as limestone (Mitchell & Gu, 2000) and concrete (Giannantonio, Kurth, Kurtis, & Sobecky, 2009). Finding from frescoes and masonry in a Russian cathedral showed that the autotrophic, nitrifying bacteria were found to be the first colonizer on frescos. Heterotrophic microorganisms including bacteria and fungi grew and utilized the cellular components of the first colonizers, this hypothesis was supported by the finding that most of the heterotrophs were able to hydrolyze bacterial and yeast cell walls (Bock et al., 1988; Karpovich‐Tate & Rebrikova, 1991). Although there was a high diversity of bacteria, the majority of sequence reads belonged to *Bacillus* and *Cellulosimicrobium* spp. Thus, the community was dominated by a relatively limited set of bacteria. The community sequencing data shows that four bacterial groups dominated both sites, which was supported by the microscopy images indicating that the communities are not morphologically diverse. FISH‐based analysis suggested that the fungi were the more dominant group based on overall biomass. Hence, the bacteria may not be the primary colonizers of the roof surfaces.

It was observed here that the microbial communities found on the Burrawang panels fluctuated seasonally, with a maximum 28 day mean of 27°C in mid‐summer and a minimum mean of 11°C in mid‐winter. This was supported by one study showing that indoor and outdoor fungi varied according to season, with a maximum count in summer when *Cladosporium* sp. was the most abundant, compared to *Penicillium* sp. and *Aspergillus* sp. that were the dominant organisms in winter (Medrela‐Kuder, 2003). In contrast, the samples from Malaysia did not show any apparent differences throughout the year possibly owing to the relatively constant temperatures in Kapar throughout year (27–30°C). While water is required for growth, our data suggested that there was no correlation between community composition and rainfall and this may suggest that water was not a limiting factor given the relatively high rainfall throughout the year.

The roof panels represent a harsh environment, where microorganisms have to contend with high levels of solar radiation, high temperature, intermittent water availability, desiccation, and relatively low nutrient inputs. However, despite these challenges, the panels were relatively heavily fouled by the end of the experiment, and the biomass of the fouling community showed a steady increase over time, suggesting that the communities were viable. This survival may be a consequence of adaptation to these habitats, where such communities would experience extended periods of dry weather (Burrawang in particular), suggesting they must be capable of surviving desiccation stress, possibly through production of osmotic protectants, such as osmolytes (Gaylarde & Gaylarde, 2005), as well as biofilm matrix components (Flemming & Wingender, 2010). Biofilms consist of a consortium of microorganisms such as bacteria and fungi settling in a matrix of extracellular polymeric substances (O'Toole, Kaplan, & Kolter, 2000). The individual cells in the biofilms are protected by their special biofilm structures from environmental stresses such as UV, desiccation (Costerton, Lewandowski, Caldwell, Korber, & Lappin‐Scott, 1995; Morris & Monier, 2003).

*Bacillus* species often dominate in soil and painting environments, and their survival could be explained by their ability to form spores (Gorbushina et al., 2004). It is therefore not surprising that we also observed a relatively high proportion of *Bacillus* species in the community sequence data, although it remains possible that they are inactive on these surfaces. Finally, it is not possible to rule out that some of the OTUs observed derived from dead cells, where the residual DNA was extracted. Thus, the low abundance organisms could represent those that have been transported, e.g., by wind or rain, to the panels, but were incapable of growth. Further work will be required to determine what proportion of the low abundant organisms are viable and capable of growth.

This study provides valuable information on the microbial composition and changes from the roof panels and demonstrated that fungi and bacteria are found in all samples but that the overall community diversity is low, where such communities were typically dominated by six organisms representing 99% of the entire community. The fungal communities were initially colonized and dominated by *Cladosporium* sp., *E. nigrum*, and *Aureobasidium* sp. followed by a change in community composition, what was dominated by Teratosphaeriaceae sp. during the later stages at Burrawang. A replicate exposure study 1 year later showed similar fungal communities colonized the painted steel coupons, while in contrast, there were differences in the bacterial communities between the two replicate studies. These results suggest that a few fungi grew on the panels as the main active organisms, and therefore, the strategies to fouling control on these roofing materials would be related to limiting the food source and control these active organisms.

## Conflict of Interest

None declared.
