# Supplementary material for: Investigation of the microbial communities colonizing prepainted steel used for roofing and walling
Source: Microbiologyopen. 2016 Dec 20;6(2):e00425. doi: 10.1002/mbo3.425 (PMC5387312; doi:10.1002/mbo3.425)
Supplement: Supplementary file 1 [file MBO3-6-na-s001.docx]

Supplementary Tables and Figures

Table S1. BLAST analysis of the amplified and sequenced 16S rRNA gene or ITS of isolates from Burrawang panels.

| **Isolate** | **Number of nucleotides** | **Identity** | **Domain** | **Accession number** | **% Identity** |
| --- | --- | --- | --- | --- | --- |
| 1 | 576 | *Epicoccum nigrum* | Eukarya | [FJ904918.1](http://www.ncbi.nlm.nih.gov/nucleotide/241017503?report=genbank&log$=nucltop&blast_rank=2&RID=TVDK2ZW6012) | 100 |
| 2 | 572 | *Cladosporium cladosporioides* | Eukarya | [GU566222.1](http://www.ncbi.nlm.nih.gov/nucleotide/291498410?report=genbank&log$=nucltop&blast_rank=57&RID=U1FZAXT701N) | 100 |
| 3 | 587 | *Alternaria sp.* | Eukarya | [GU584951.1](http://www.ncbi.nlm.nih.gov/nucleotide/315435165?report=genbank&log$=nucltop&blast_rank=1&RID=TVEDCCR2012) | 99 |
| 4 | 188 | *Micrococcus sp.* | Bacteria | [JQ229722.1](http://www.ncbi.nlm.nih.gov/nucleotide/383850162?report=genbank&log$=nucltop&blast_rank=2&RID=TVERY3ED016) | 98 |
| 5 | 150 | *Spirosomarigui sp.* | Bacteria | [AB681384.1](http://www.ncbi.nlm.nih.gov/nucleotide/359805017?report=genbank&log$=nucltop&blast_rank=1&RID=TVEYA3N001S) | 99 |
| 6 | 582 | *Cladosporium sp.* | Eukarya | [HQ631003.1](http://www.ncbi.nlm.nih.gov/nucleotide/317383324?report=genbank&log$=nucltop&blast_rank=2&RID=U1J658FA016) | 99 |
| 7 | 603 | *Stagonospora sp.* | Eukarya | [AJ496626.1](http://www.ncbi.nlm.nih.gov/nucleotide/27529037?report=genbank&log$=nucltop&blast_rank=1&RID=TYRWWBT3016) | 99 |
| 8 | 202 | *Hymenobacter sp.* | Bacteria | [EF423320.1](http://www.ncbi.nlm.nih.gov/nucleotide/126567812?report=genbank&log$=nucltop&blast_rank=31&RID=TYSTE9J0016) | 99 |
| 9 | 127 | *Sphingopyxis sp.* | Bacteria | [AB235163.1](http://www.ncbi.nlm.nih.gov/nucleotide/75812142?report=genbank&log$=nucltop&blast_rank=4&RID=TYU1453X01S) | 99 |
| 10 | 135 | *Pseudomonas aeruginosa* | Bacteria | [GU294304.1](http://www.ncbi.nlm.nih.gov/nucleotide/281334112?report=genbank&log$=nucltop&blast_rank=3&RID=TYUXWD7101N) | 96 |
| 11 | 586 | *Alexandrium catenella* | Eukarya | [JF343259.1](http://www.ncbi.nlm.nih.gov/nucleotide/324962911?report=genbank&log$=nucltop&blast_rank=1&RID=TYV8TCYC013) | 95 |
| 12 | 479 | *Fusarium pseudograminearum* | Eukarya | [DQ459871.1](http://www.ncbi.nlm.nih.gov/nucleotide/91719253?report=genbank&log$=nucltop&blast_rank=1&RID=Y26SFAAU01S) | 86 |

Table S2. BLAST analysis of PCR amplified and sequenced 16S rRNA genes or ITS of isolates from Kapar panels.

| **Isolate** | **Number of nucleotides** | **Identity** | **Accession number** | **% Identity** |
| --- | --- | --- | --- | --- |
| M1 | 544 | *Hypoxylonmonticulosum* | GQ999283.1 | 99 |
| M2 | 483 | *Periconiamacro spinosa* | JX427048.1 | 96 |
| M3 | 456 | *Aspergillus tubingensis* | KP418575.1 | 100 |
| M4 | 590 | *Cryptococcus sp.* | KM587000.1 | 100 |
| M5 | 527 | *Aureobasidium pullulans* | KM044085.1 | 100 |
| M6 | 514 | *Nigrospora sphaerica* | KM893076.1 | 100 |


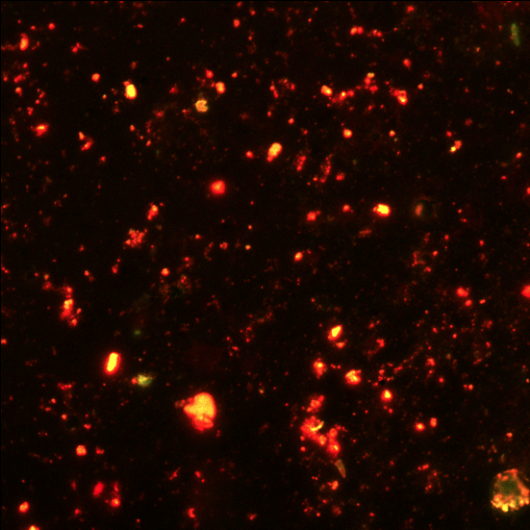

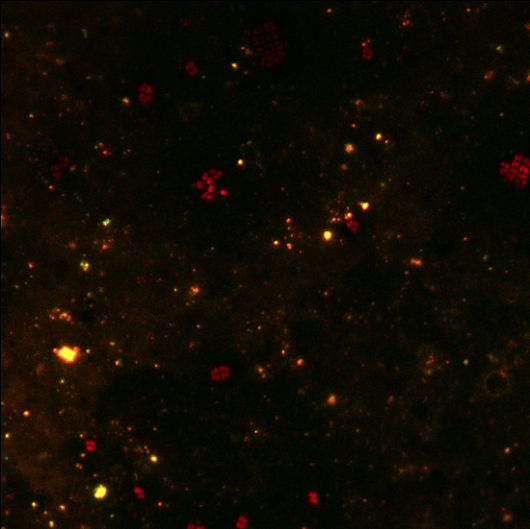


fungi

bacteria

fungi

bacteria

B

A

Figure S1. Detection of bacteria and fungi by fluorescence in situ hybridization (FISH). Bacteria were hybridized with the EUB-Cy3 probe (green) and fungi were hybridized with the EUK-Cy5 probe (red). Images were collected from painted steel panels that had been exposed for (A) 4 weeks and (B) 24 weeks at Burrawang. Scale bar =50 µm.


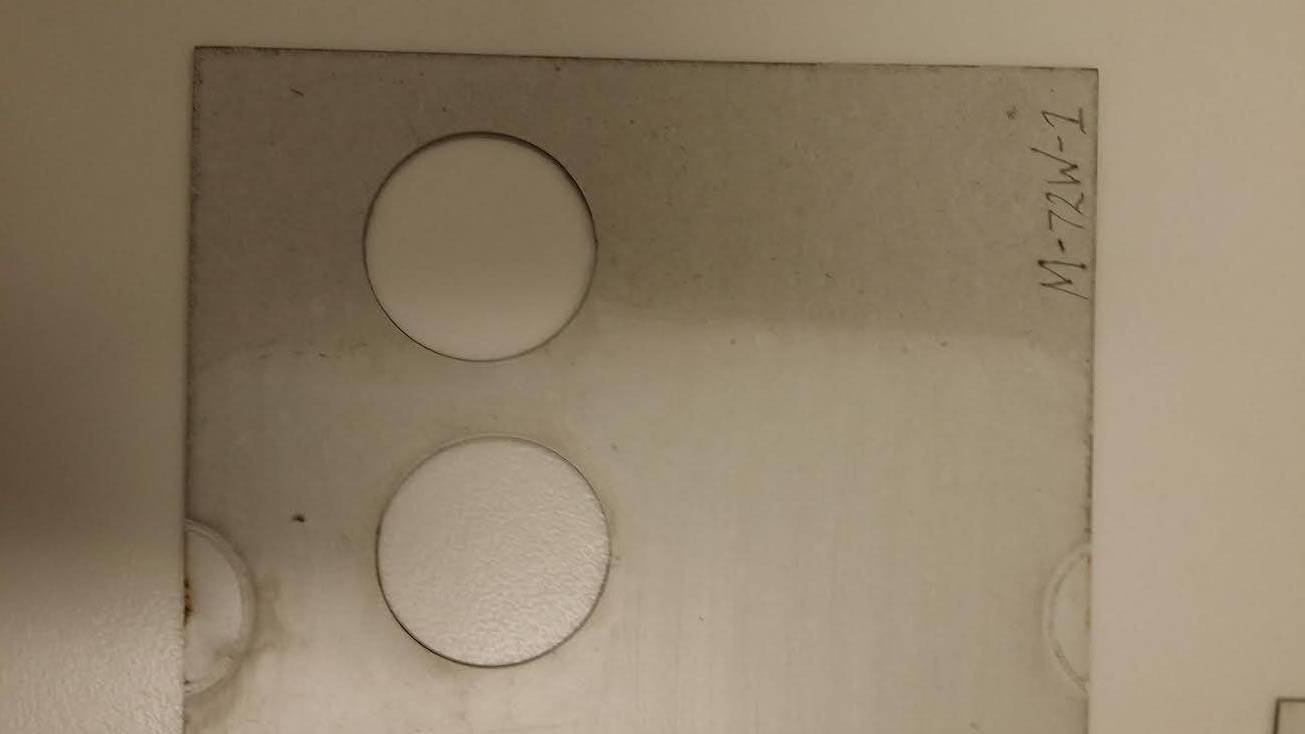


Figure S2. A representative panel, collected in Kapar after 72 weeks incubation, showing un-swabbed (top) and after swabbed (bottom). The circle indicated (🡪) is where the coupon was fixed onto the exposure rack by a rubber washer (and hence is unfouled).

**Calculation of richness and evenness**

For community sequencing data, biodiversity, measured by species richness and the Shannon diversity index, was calculated using Qiime and is represented as rarefaction curves. Data are presented based on the average of duplicate biological samples.

B

A


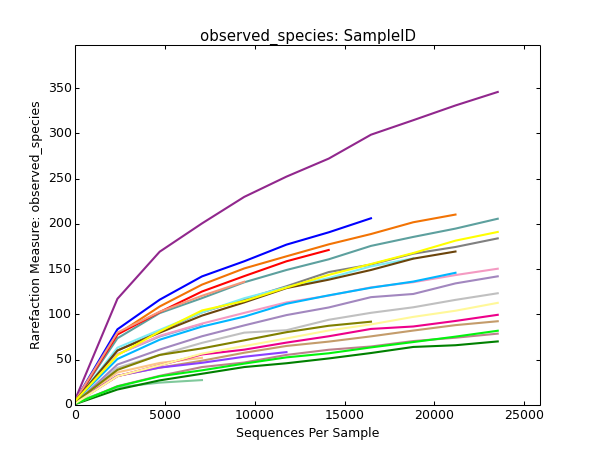

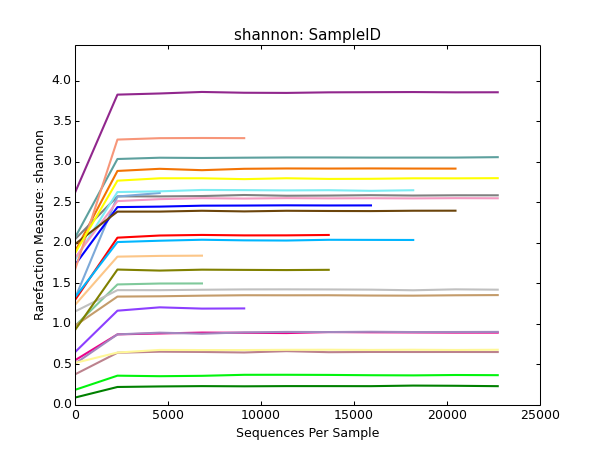


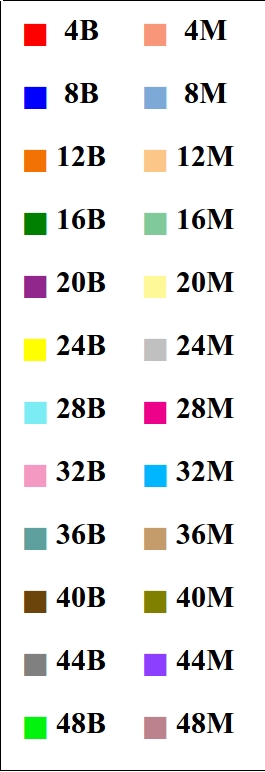


**4K**

**8K**

**12K**

**16K**

**20K**

**24K**


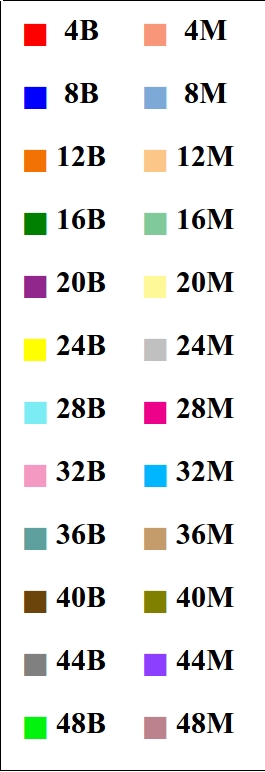


**28K**

**32K**

**36K**

**40K**

**44K**

**48K**


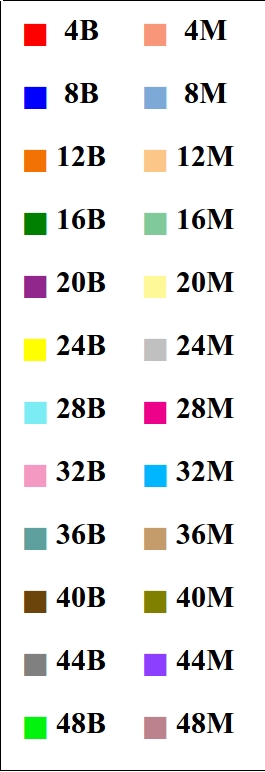


**28B**

**32B**

**36B**

**40B**

**44B**

**48B**


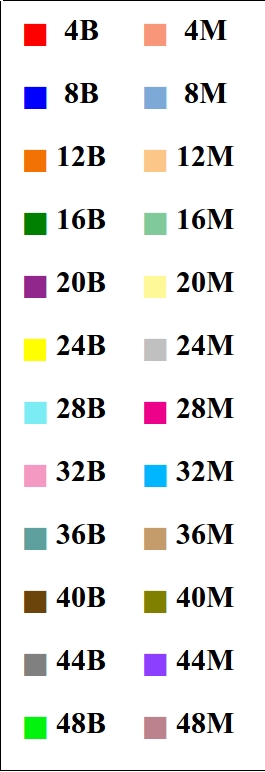


**4B**

**8B**

**12B**

**16B**

**20B**

**24B**

D

C


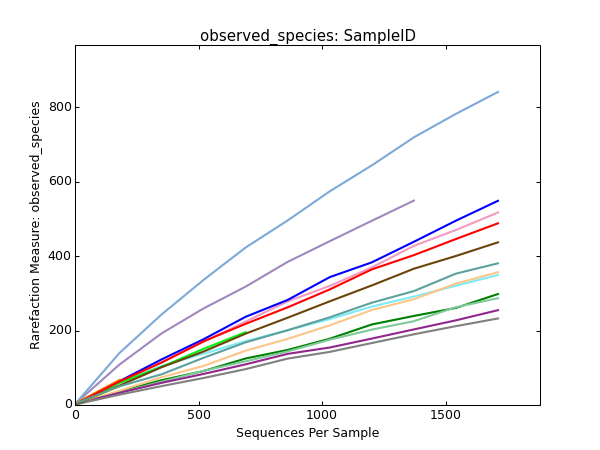

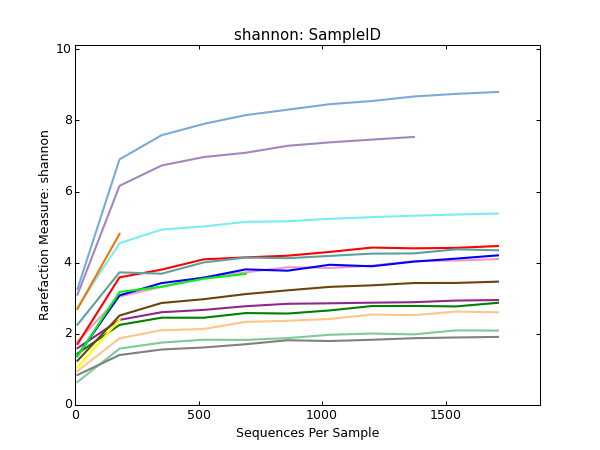


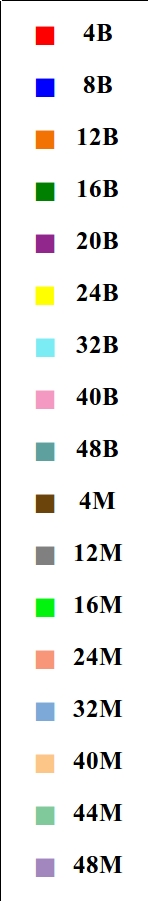

**4K**

**12K**

**16K**

**24K**

**32K**

**40K**

**44K**

**48K**

Figure S3. Biodiversity (Shannon index (B, D)) and Richness (observed species, A, C) of fungal (A, B) and bacterial (C, D) communities from painted steel surfaces from Burrawang and Kapar. Richness and biodiversity were calculated using Qiime based on community sequencing data targeting the bacterial 16S rRNA gene or the fungal ITS region. B=Burrawang, K=Kapar

Size of terminal restriction fragments (bp)


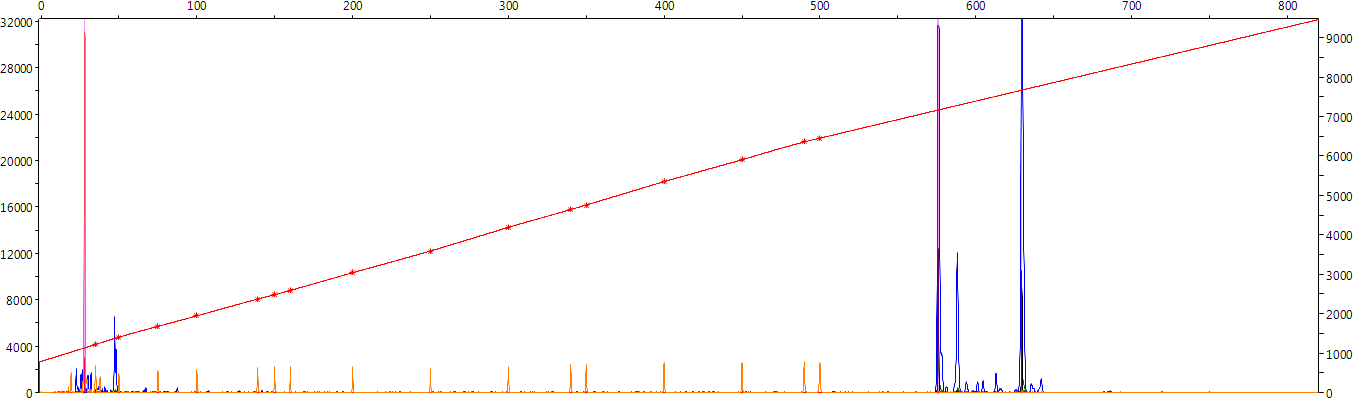


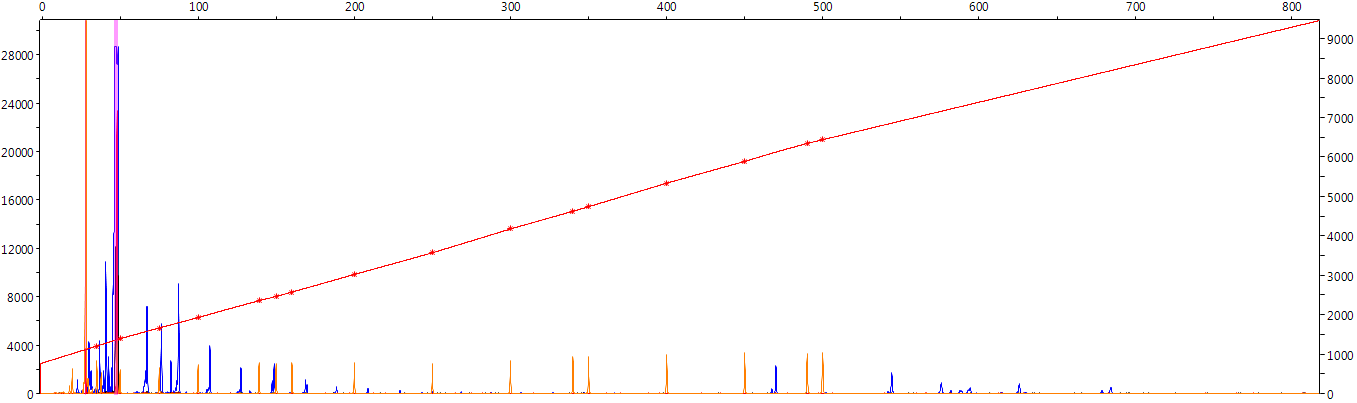


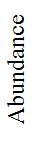


Figure S4. A representative T-RFLP profile from coupons incubated for 48 weeks at Burrawang (A) and Kapar (B). The red line represents control size standards.
